# Eigenmodes of the deep unconscious: the neuropsychology of Jungian archetypes and psychedelic experience

**DOI:** 10.1093/nc/niaf039

**Published:** 2025-10-18

**Authors:** Hugh McGovern, Marco Aqil, Selen Atasoy, Robin Carhart-Harris

**Affiliations:** School of Medicine, Deakin University, Health Education Research Building (HERB)—Level 3, Barwon Health, P.O. Box 281, Geelong, VIC, 3220, Australia; McElwain Building (24A) The University of Queensland St Lucia QLD 4072, Australia; The Cairnmillar Institute, Level 1, 391-393 Tooronga Road, Hawthorn East, VIC 3123, Australia; Independent Researcher, Amsterdam, Netherlands; Centre for Eudaimonia and Human Flourishing, Linacre College, University of Oxford, Stoke House, 7 Stoke Place, Oxford OX3 9BX, United Kingdom; Departments of Neurology and Psychiatry, The University of California San Francisco, 675 18th Street San Francisco, CA 94143, United States

**Keywords:** psychedelics, Jung, archetypes, predictive processing, cognition, beliefs

## Abstract

This article presents a neuroscientific interpretation of Carl Jung’s theory of archetypes and their experience in altered states of consciousness. We begin by rehearsing the Free Energy Principle and Predictive Processing as foundational frameworks that subserve and inform the thesis that follows. The following sections examine three aspects of archetypes: the affective core rooted in subcortical systems, archetypal imagery emergent in altered states such as psychedelic experiences, and archetypal stories encoded in higher cortical areas. Specifically, we propose a trilogical interplay between the high-level cortex, the low-level cortex, and subcortical/affective systems in instantiating these archetypal phenomena. We then explore how archetypes may be transmitted between individuals, developing into a collective unconscious through social learning and subsequent attunement. Throughout, we provide syntheses of Jungian psychology with contemporary neuroscience, offering testable hypotheses regarding the neurological bases of archetypal phenomena. We conclude by discussing implications for both psychoanalytic theory and neuroscientific research. By bridging these disciplines, we aim to lend construct validity to Jungian concepts and encourage further empirical investigation of archetypes and the collective unconscious.

## Introduction

In this article, we address the problem of how the Jungian construct of archetypes can be understood from a neuroscientific perspective. We parse the concept of archetype into distinct categorizations, arising from discrete cortical and subcortical circuits. Echoing Jung, we distinguish between the “archetype as such,” the “archetypal image,” and the narrative or symbolically based “archetypal story.” Archetypes ‘as such’ refer to the tendency to form particular predictions, or the recurrent brain-based patterns that give rise to the archetypal ‘images’ and ‘stories’. The archetypal ‘image’ is the sensory representation or manifestation of the archetype as such, and the archetypal ‘story’ refers to the narrative form. We suggest that the archetypal ‘core’ is primarily affective; this ‘core’—close but not quite identical to the archetype ‘as such’—becomes clothed in sensory aspects (the archetypal experience in prereflexive consciousness); and finally reaches highest levels of the cortex where it takes narrative form (the archetypal ‘story’, or the rational, conscious conceptualization of the archetype). We principally refer to the archetypal ‘image’ outlined above but note when we discuss other archetypal instantiations.

We consider each instantiation of archetypes as arising from fundamental patterns, or semantic themes, principally expressed and depicted through interplay between the visual modality (i.e. as “archaic images”), low-level cortex, and affective/subcortical systems (see below for further discussion. The resultant archetypal patterns and themes transcend the individual (i.e., they are universal, shared, or collective), and are multi-scale: (i.e., they apply both at a low (e.g., sensory visual, in some cases geometric)), and high, abstract level (such as benevolent or malevolent others). In keeping with the complexity of the issues at hand, our cardinal aim is to raise questions on the processes by which phenomenological experiences of archetypes arise. We thus note that we do not seek to provide a definitive framework under which archetypes must be understood. Rather, we seek to understand and discuss the neural correlates, primarily of archetypal images, or the experience of archetypes, such as when one enters altered states of consciousness.

We further suggest that advances in the neurosciences enable planning of experiments that could test key tenets of Jungian psychology, especially as they pertain to the construct of archetypes. We take inspiration from the complementary frameworks of the Bayesian Brain, Hierarchical Predictive Processing, and the Free-Energy Principle (Friston 2010) and we consider psychedelic (translated “psyche revealing”) drugs as powerful tools for probing the target phenomena of interest. Specifically, we propose that archetypes can be understood as shared minima, evinced by regular or stable spatiotemporal patterns that encode sufficiently stable patterns or themes across conspecifics. That is, for the experience to be shared, we assume that the specific properties of its brain instantiation must be sufficiently conserved or consistent across individuals. In other words, there must be a shared (neural and cultural) basis for specific archetypal experiences (i.e. archetypes as such).

Although the task won’t be easy, we argue that the empirical work we consider could lend ‘construct’ validity to the Jungian notion of the “collective unconscious” where archetypes are a special property of this that can be evoked and examined. Validating constructs from psychoanalytic psychology (e.g. by demonstrating the shared biological basis of shared archetypal *images* or visions) will have broad and specific implications. These implications include increasing the validity of psychoanalytic psychology and the utility of psychedelic drugs as experimental tools for inducing the emergence of relevant phenomena. Moreover, validation of theoretical principles from depth psychology lends a scientific grounding to some of the guiding assumptions of psychoanalytic psychotherapy. Lastly, the work we propose will have implications for competing epistemologies, for example, by demonstrating the biological (brain) basis of exceptional or so-called “mystical” experiences (Romeo et al. 2021). Below, we situate our discussion by rehearsing the fundamental assumptions of the Free Energy Principle (FEP) and Predictive Processing. We then move to discussion of the affective and subcortical/limbic basis of archetypes as such, archetypal imagery, and how psychedelic effects solicit archetypal imagery (i.e. letting off the leash, see p. 9), before finishing with discussion of how such archetypal instantiations flow between conspecifics. We conclude with offering implications of this novel synthesis.

Before proceeding with rehearsal of key neuroscientific concepts, we will briefly orient the reader to core Jungian concepts. Jung posits that the psyche consists of both interpersonal and transpersonal aspects. Most relevant for our purposes is the notion of the collective unconscious, a transpersonal aspect of the psyche in which lie archetypes, defined broadly as innate and preconceptual forms that provide shape to emotion, perception, and meaning (Jung 1959). Importantly, archetypes are proposed to manifest in several ways, including in symbolic motifs, mythology, imagery or patterns, or altered states of consciousness. Jung notes that archetypes are not reducible to specific contents (i.e. there is no one instantiation of an archetype) but instead reflect recurring propensities toward how subjective experience is organized, which Jung has referred to as typified modes of apprehension (Jung 1959).

In what follows, we note that our aim is not to provide neuroanatomical mappings of Jungian constructs. Rather, we seek to explore how recent advances in the neurosciences provide clarity on the neurological conditions under which archetypal content becomes more especially salient and how the hierarchically structured organization of the brain affords the emergence and experience of the collective unconscious and archetypes. We are careful to note the fact that Jungian concepts are symbolic and imaginal in nature, marking them as particularly rich. We seek to draw parallels with Jungian symbolic or imaginary language, and the mechanistic accounts of contemporary neuroscience, rather than proposing a one-to-one mapping. We thus seek to offer a ‘synthesis’, wherein Jungian thought can be meaningfully described in terms of modern neuroscience, without losing the theoretical richness of Jung’s work.

### The Free Energy Principle and predictive processing

Primarily attempting to explain imperatives for self-organizing systems, the FEP extends the Helmholtzian notion of unconscious inference (Helmholtz 1867). The FEP assumes adaptive cognitive systems instantiate hierarchically organized models to minimize variational ‘free energy’—an information theoretic term indexing divergence between predicted and sampled data in the sensorium (Friston 2010). The FEP describes how the brain resolves a fundamental imperative of biological systems, namely, to resist nature’s natural tendency toward entropy. For individual organisms and the brain, this imperative—of remaining in characteristic and sustainable states (i.e. attracting states)—translates to a recurrent (short-term) motivation to avoid surprise (or variational free energy, often cast as prediction error; Friston 2010).

#### Predictive processing

Falling under the general framework of the FEP, predictive processing suggests the brain, to attain its imperative of reducing surprise, self-organizes to match and anticipate the most likely causes of sensory data (Keller and Mrsic-Flogel 2018). Predictive processing assumes bottom–up and top–down circuits operate in terms of “message passing” to facilitate prediction or modeling of the environment. In predictive coding formulations of implicit Bayesian belief updating, ascending (bottom–up) prediction errors update representations or statistical expectations—at higher levels—to resolve prediction errors at lower levels *via* descending (top–down) predictions. (Friston 2010, Hohwy 2013). Top–down neural activity linked to re-entrant loops (i.e. recurrent message passing between hierarchical levels; see Bastos et al. 2012, Walsh et al. 2020) propagate predictions about inputs given a prior representation about its cause (Friston 2010). Conversely, ascendant signals from sensory levels propagate ensuing prediction error (Friston 2008, Friston 2010, Clark 2013). If descending predictions sufficiently account for the ascendant sensory signals, no prediction error or surprise is registered. In such cases, the prediction error is “explained away”— *via* inhibitory mechanisms—driven by descending or top–down information flow encoding “predictions” from the higher cortex (Friston 2010).

These recursive exchanges with the external milieu, and recurrent message passing in cortical circuits, facilitate the emergence of a generative world model. The hierarchical minimization of prediction error is key here, as it offers a criterion on which organisms can build and update a world model that can generate sensory predictions: allowing then to subsequently explain sensory data. This framework allows us to describe how the brain makes sense of—and resolves—ambiguous sensory data. A crucial aspect here is that relatively higher regions of this hierarchy encode more abstract information accrued over longer temporal windows, whereas lower levels encode more concrete, sensory representations accrued over shorter temporal windows (Hasson et al. 2015, Friedman and Robbins 2022).

### Predictions, ontogeny, and evolution

An important point here is that the brain, and by virtue its predictive capacities, is not formed *in vitro*, but is steadily constructed and scaffolded throughout development. To properly understand how archetypal material is constrained in hierarchical generative models (we later describe psychedelic experiences as relaxing or letting these constraints “off the leash”), we first need to situate brain development within a broader ontogenetic and evolutionarily literate framework.

The dynamic of ascending signals from subcortical structures, with accompanying constraint from descending cortical predictions, may reflect a more general neurodevelopmental and evolutionary pattern. Sterelny (2012) notes that human lineages are characterized by an unusually long ontogeny encompassing infancy, childhood, adolescence, and beyond. This extended developmental arc is important for two reasons. First, it allows an extended period of cultural scaffolding and intergenerational apprenticeship through repeated engagement with culturally specific or mediated practices such as storytelling, through which children learn to interpret emotional experience *via* symbolic narratives like myth and folklore. This supports the development of what Sterelny calls an information commons. Second, extended ontogeny introduces a developmental lag between the early emergence of affectively salient subcortical systems and the later maturation and integration of large-scale cortical networks such as the default mode and frontoparietal control networks (Fair et al. 2008, Andrews-Hanna et al. 2014, Sherman et al. 2014, Whitaker et al. 2016).

Research in developmental neuroscience shows that regulatory networks important for higher-order cognition and inhibitory control are only sparsely connected in early and middle childhood and become more coherent in late adolescence. This lag has been described as a neural maturation gap (Jones 2013), where evolutionarily ancient brain systems and more recently evolved cortical structures are not yet fully differentiated or coordinated. This gap may increase vulnerability to psychopathology but also provides a neurodevelopmental basis for what Jung referred to as the split psyche (Jung 1921/2014b). For Jung, the tension between ego consciousness and the broader collective unconscious reflected a deeper antagonism between recently evolved and more ancient brain structures (Jung 2014b, pp. 253–264). In particular, Jung suggested that ego development could create intrapsychic dissociation, soliciting conflict between what one consciously aims to be, and what one unconsciously is. Such tension, in turn, could create neuroses, but also can initiate the process of individuation when properly symbolically integrated.

Clark (2022, 2025) develops this insight further, arguing that Jung’s psychic antagonism maps onto the asynchronous emergence of subcortical and cortical systems. From this perspective, the split psyche reflects the structure of human ontogeny, specifically the temporal gap between early developing affective systems and later developing regulatory systems. Clark (2025) further suggests that cultural practices including ritual, myth, and altered states of consciousness may be understood not just as social tools but as symbolic strategies evolved to manage such mismatch. For example, rites of passage do not just transmit cultural norms but facilitate symbolic reconciliation between unconscious material and ego consciousness. This interpretation resolves a puzzle: if altered states are evolutionarily useful, why do they suppress the cortical systems central to human cognition? Clark notes that such states are not just adaptations in the evolutionary sense but rather culturally evolved responses to developmental tensions between evolutionary more ancient affective circuits and newer regulatory cortical systems. Jung anticipated this idea in his description of ritual as a symbolic vessel that supports psychic integration and individuation (Jung 2014a, Jung 1921).

The metaphor of being off the leash, developed in what follows, reflects this developmental architecture. Psychedelic states, for example, may briefly disrupt the gating effects of cortical maturation (Carhart-Harris and Friston 2019), permitting access to emotional and archetypal material that is normally inhibited in maturity. Such states do not just disrupt cortically instantiated prediction but also reveal earlier stages of psychological organisation. In this view, ontogenetic delays in brain development and their symbolic resolution through cultural forms are crucial to understanding the reemergence of archetypal content in altered states of consciousness.

## The brain, prediction, and archetypal modes of representation

The predictive processing paradigm and accompanying ontogenetic and evolutionary frameworks situate the thesis we develop here. Namely, we propose that archetypes ‘as such’ and archetypal ‘images’ are instantiated *via* a prediction cascade over various cortical and subcortical systems. Crucially, we posit that these notions of archetypes (i.e. archetypes ‘as such’, archetypal ‘images’, and archetypal ‘stories’) are instantiated *via* a “trilogical interplay” involving the high-level cortex, the low-level cortex, and subcortical/affective systems. Below, we review evidence for how each of these systems supports instantiating archetypes ‘as such’, archetypal ‘images’, and archetypal ‘stories’, before turning attention to modes of archetypal transmission.

### The affective core and archetypes

The first component of our thesis concerns the affective core of archetypes. Jung considered the subcortical systems of the brain as the physiological bases for archetypal emergence: characterized by an affective core (Jung 1958).

*I have long thought that, if there is any analogy between psychic and physiological processes, the organizing system of the brain must lie subcortically on the brain stem. This conjecture arose out of considering the psychology of an archetype [the Self] of central importance and universal distribution represented in mandala symbols. … The reason that lead me to conjecture a localization of a physiological basis for this archetype in the brain stem was the psychological fact that besides being specifically characterized by the ordering and orienting role, its uniting properties are predominantly affective. I would conjecture that such a subcortical system might somehow reflect characteristic of the archetypal form of the unconscious.—*(Jung 2014, para 582).

This supposition is supported by contemporary neuroscience. Subcortical midline structures have recently been referred to as the affective core of the self, and a primary filter through which objects of consciousness are interpreted (Alcaro et al. 2017, (Solms and Friston 2018, Solms 2021). Such structures are fundamental processes including regulation of visceral and somatic states (Damasio 1996, 1999, Porges 2011), orienting movements (e.g. survival stances, sexual behavior) (Tinbergen 1951, Denton 2006), and integration of sensory experiences into affective value (Merker 2007) (see Alcaro et al. 2017, for overview). This has led to speculation that subcortical midline structures serve as a fundamental basis of primordial subjectivity in humans and animals (Panksepp 2016, Seth et al. 2005, Northoff and Panksepp 2008, Edelman and Seth 2009, Panksepp and Northoff 2009, Revonsuo 2009, Ward 2011, Panksepp and Biven 2012, Fabbro et al. 2015, Feinberg and Mallatt 2016). This notion is reflected in Jung’s perspective of the mind as hierarchical and proposed to be supported by a hierarchical neural architecture (Alcaro et al. 2017).

A crucial aspect of the thesis forwarded here is that these subcortical and limbic systems must project predictions into cortical structures, so to facilitate the emergence of subsequent collating of information that supports archetypal imagery. At the level of the brain, this necessitates predictions regarding the self and the world, fed from subcortical and limbic regions (Joseph 1992) to cortical regions, to integrate affectively based predictions into a kind of general representation instantiated by cortical structures (Hansen et al. 2024). This is supported by recent work (Siman-Tov et al. 2019, Clark et al. 2021, Ficco et al. 2021). Siman-Tov et al. (2019) found evidence of a ‘prediction network’ involving directed predictions from subcortical structures which enact influence on how cortical structures integrate sensory information. Subcortical regions such as the thalamus

Sherman and Guillery 2006, Halassa and Kastner 2017, particularly the pulvinar (Saalmann and Kastner 2011) can mediate the influence of top–down cortical predictions, and transmit predictions to cortical regions (Kanai et al. 2015). Solms 2019, Solms 2021) formulates the influence of certain subcortical structures as equipping cortical prediction errors with the right precision, through ascending (classical) modulatory projections. In this sense, this influence underwrites affect and anxious feelings as a form of “felt uncertainty” (e.g. see McGovern et al. 2022) (NB, precision is the counter-weight of uncertainty) (Solms 2018, Solms 2021).

### Synthesis

Jung suggested archetypes likely emerge from subcortical regions, particularly the brain stem, and are likely characterized by an affective core. The evidence above points to an organizing and central role of subcortical systems in subserving subjectivity and projecting predictions—including predictions of precision—to a hierarchically higher cortex. Subsequent representations in the cortex are thus infused with this affective undertone. This is supported by comparative and neuroscientific work, which suggests that subcortical structures, particularly midline subcortical structures, may serve as an affective core, thence serving as a fundamental basis of primordial subjectivity in humans and animals alike.

### The archetypal image and altered states

Jung distinguished between archetypes ‘as such’ (tendency to form predictions, and stemming from subcortical regions) and archetypal ‘images’. These images were proposed to be the sensory instantiations of archetypes ‘as such’ and are often experienced in altered states of consciousness, such as those solicited by psychedelic drugs and in the dream or dreamlike states (Carhart-Harris 2007). Subjective reports of archetypal ‘images’ (in the Jungian sense) are a consistent theme in psychedelic and dream states (Masters and Houston 2000; Shanon 2002, Lawrence et al. 2022). This is consistent with much of what Jung argued, namely, that the unconscious houses psychological remnants of phylogenetic ancestry (Carhart-Harris 2007, Carhart-Harris et al. 2014). Under psychedelics, these often latent and recurrent themes become manifest in conscious experience. This is consistent with the phenomenology of psychedelic experiences, where people often report encounters with archetypal patterns and beings (e.g. *feminine archetypes, divine beings*). Jung states:

*“Experiments along the line of mescaline and related drugs are certainly most interesting, since such drugs lay bare a level of the unconscious that is otherwise accessible only under peculiar psychic conditions. It is a fact that you get certain perceptions and experiences of things appearing either in mystical states or in the analysis of unconscious phenomena”—*  Jung 2015, p. 382.

The phenomenological alterations under psychedelics are thought to be because serotonergic psychedelics are 5-HT_2A_ agonists. 5-HT_2A_ receptors mediate neuromodulatory changes in postsynaptic gain (i.e. modulate precision) and are densely expressed in domain-general regions of cortex such as not only the high-level cortex, including the default mode network (DMN), but also the primary visual cortex (V1) (Beliveau et al. 2017, Carhart-Harris and Friston 2019). The affinity of serotonergic psychedelics toward 5-HT_2A_ receptors—densely expressed in the high-level cortex—is thought to result its functional disintegration (a.k.a. loss of intra-network functional connectivity), with concomitant increased connectivity between networks (Lebedev et al. 2015, Tagliazucchi et al. 2016, Girn et al. 2023). In other words, regions of high-level cortex become less entraining of lower-level regions and more sensitive to ascending inputs (c.f. ascending prediction errors from hierarchically lower levels). In the acute phase, psychedelics decrease functional connectivity in intrinsic networks, and increase between-network cross-talk (Roseman et al. 2014, Lebedev et al. 2015, Palhano-Fontes et al. 2015, Carhart-Harris et al. 2016, Nichols 2016, Tagliazucchi et al. 2016, Atasoy et al. 2017, Müller et al. 2018, Mason et al. 2020, Mertens et al. 2020, De Gregorio et al. 2021, Grandjean et al. 2021, Daws et al. 2022, Madsen et al. 2021).

Moreover, insofar as psychedelics increase between-network connectivity, they alter consciousness experience (Letheby and Gerrans 2017, Müller et al. 2018, Smigielski et al. 2019, Mason et al. 2020, dos Santos and Hallack 2021, Daws et al. 2022). As such, the acute phase of the psychedelic experience is thought to be characterized by a sharp departure in brain dynamics: from a modular and functionally segregated organization to more chaotic and distributed neuronal dynamics (Palhano-Fontes et al. 2015, Smigielski et al. 2019, Girn et al. 2023).

With the classic psychedelics, visual imagery or hallucinations during the acute psychedelic experience seemingly result from the impact of activation of 5-HT_2A_ receptors. This activation, in turn, enhances excitability of layer 5 pyramidal cells in the cortex, facilitating increased glutamate release (Nichols 2016, Carhart-Harris and Friston 2019, Mason et al. 2020). Activation of cortical 5-HT_2A_ receptors results in asynchronous glutamate release, causing subsequent asynchronous activity in neural ensembles (Riba et al. 2006, Celada et al. 2008, Nichols 2016, Tagliazucchi et al. 2014). Spontaneous ensemble activity subsequently becomes more asynchronous, with the end results being a loss of synchronous gain (Aghajanian and Marek 1999, Tagliazucchi et al. 2014). See Chawla et al. (1999) for a mechanistic discussion of this neuromodulatory effect. These disruptions translate across scales [i.e. at the initial level of a spike-to-wave decoupling, then ensemble-level fields, network “disintegration,” and whole-brain level network desegregation (Nutt et al. 2020, Girn et al. 2023)].

Perceptually, this “entropic” and “anarchic” quality of brain function is hypothesized to parallel a decreased precision of predictive mechanisms (predictions are referred to as “priors” in Bayesian nomenclature) such that, for example, precepts are less stable and constrained. An increased sensitivity to ascending afferents is a corollary of the relaxation of top–down predictive mechanisms. Also in parallel, an excitation (Aqil and Roseman 2023) of lower levels of the functional hierarchy (e.g. the visual cortex) likely drives ascending information flow from the visual cortex up the visual hierarchy. This mechanism may encode reports of increased information content in conscious experience (Timmermann et al. 2023)—also scored as increased psychological insight (Lyons et al. 2024). In one model, the relaxation of entraining and constraining top–down information flow, combined with a direct excitation of visual regions in particular, driving ascending information flow (Aqil and Roseman 2023) could account for the arising of archetypal ‘images’ such as patterns and beings under psychedelic drugs, where “information” carried by excitation of an organized or structured visual cortex is allowed to propagate (Ermentrout and Cowen 1979). Some recent data suggest that this manifests as higher spatiotemporal frequency patterns or harmonics on the cortical surface and across the whole brain (Atasoy et al. 2016, Atasoy et al. 2017). Harmonics here refer to spatiotemporal patterns of activity arising from the brain’s connectome. As musical harmonics can describe the vibrations of a violin string, harmonics refer to the patterns of how brain activity propagates across the cortex. These connectome harmonics thus come to resemble a kind of base set of possible states the brain can inhabit, with higher-frequency harmonics corresponding to more complex and detailed activation patterns (Atasoy et al. 2017).

With respect to archetypes, it is reasonable to posit that a flattening of the implicit free energy landscape—mediated by a loss of synchronous gain and precision in the (otherwise entraining) descending information flow (Girn et al. 2022, Singleton et al. 2022)—could enable the arising of (unstable) motifs or images encoded in ascending information flow and ordinarily latent or suppressed. These motifs are ordinarily precluded from conscious experience (due to the constraining and entraining influence of precise (heavily weighted) descending activity from higher levels of the cortex onto lower levels (Girn et al. 2022). The hypothesized “release” *via* decreased precision-weighting of priors (Carhart-Harris and Friston 2019) and direct excitation of lower sensory representations (Aqil and Roseman 2023) may cause archetypes to enter conscious awareness, i.e. as archetypal imagery, patterns, and symbolism (Szára 2014, Gallimore and Luke 2015), analogous, perhaps, to a projector projecting (affect-laden) visual information onto a screen (Winkelman 2018).

In phenomenological terms, archetypal visions (e.g. ‘entity encounters, sages, feminine phenotypes, processes of transformation’) under psychedelics may arise due to: (i) indirect emancipatory effect through dysregulated activity in the high-level cortex (Carhart-Harris and Friston 2019) and (ii) a direct excitation effect within visual cortex (Aqil and Roseman 2023) causing distinct spatiotemporal patterns (encoding representations) to resonate throughout the brain’s functional hierarchy. We do not discount a role for medial temporal structures, and the ventral visual stream in particular, performing a similar role, i.e. high-frequency spatiotemporal patterns or “harmonics” traversing the visual hierarchy, connecting V1 to the higher-level medial temporal regions (see Carhart-Harris 2007). Empirically, one might predict a “flip” in the directionality of traveling waves between these hierarchical levels under a psychedelic, such that there is less top–down and more bottom–up information flow, as seen previously in two independent studies with DMT (Alamia et al. 2020, Timmermann et al. 2023). It is curious to hypothesize that this phenomenon may differentiate the visually rich DMT experience from the typically less visual 5-MeO-DMT experience, the latter being an atypical psychedelic apparently without an appreciable 5-HT_2A_ agonist action.

Phenomenological accounts of archetypal beings (e.g. sages, witches, angels) and processes (e.g. rites of passage/coming of age, hero’s journey) could therefore reflect the outlay of innate (complex) spatiotemporal patterns underlying recurrent social cognitive scripts and representations of ancestral social life (McGovern et al. 2025) that are conserved across individuals within our species. As such, experiences of archetypes under psychedelic drugs may be a product of the human brain representing recurrent social motifs and themes accumulated by sensory and affectively charged encounters and stored, represented, and then released by liberated information flow under specific conditions. Almost by default, these would reflect the kinds of recurrent adaptive challenges and motifs (e.g. transformation or coming of age processes) and representations that were salient for a successful navigation of ancestral social life (e.g. protector figures, the mother archetype) (Neumann 2015).

### Synthesis: psychedelic brain effects and archetypal representation

We have thus far offered a synthesis and explanation of how the neurobiological effects of psychedelic drugs may relate to the phenomenological experience of archetypal (entity) encounters often reported under psychedelic drugs. Psychedelics increase the entropy of spontaneous cortical activity (Schartner et al. 2017) and disrupt hierarchical information passing in the acute phase of a psychedelic “trip” (Carhart-Harris and Friston 2019). This is consistent with the notion of hierarchically higher regions losing their characteristic constraining and entraining influence on lower-level sensory systems. In turn, this could mean that the archetypal imagery and simulations latent in the brain can be “liberated” under such conditions (i.e. archetypes as such in subcortical/limbic regions, archetypal stories in the default mode network, and archetypal images in the visual cortex). This effect may be dose-dependent: where higher doses are required to engage and disrupt the deepest aspects of the hierarchy, while lower doses may favor the direct excitation of low-level aspects (Aqil and Roseman 2023), which encode more elemental features, such as spatial geometries, motion, and color (Ermentrout and Cowan 1979).

Psychedelics could thus enable the brain to liberate archetypal simulations, causing a liberated or anarchic quality to message passing (Alamia et al. 2020) (we note this would be dependent on affective structures first being engaged first, projecting to the higher cortex, which then descends predictions to the sensory cortex). We suspect that this may manifest as an increase in spatiotemporally finer-grained brain activity patterns or “harmonics” and a decrease in coarse-grained (information constraining or compressing) patterns (Atasoy et al. 2016; 2017; Siegel et al. 2024). Thus, the relinquishment of typical top–down, hierarchical causation in the brain and the liberation of bottom-up flow may mean that phenomenological (archetypal) visions can shimmer into perceptual awareness, as representations of recurrent motifs from our shared ancestral environments and encounters as human animals. Having discussed the neurological basis of archetypes ‘as such’, and archetypal ‘images’, we now attend to the ways in which archetypal ‘stories’ come to be represented and transmitted between conspecifics.

### From the personal to the collective: the collective unconscious and modes of transmission

Consistent with his predecessor and mentor—Freud—Carl Jung argued that cognition could be parsed into different subtypes such as primary and secondary processing. Secondary processing refers to cognition of modern humans in a standard waking state, and the former being an archaic style of cognition, now only entered during altered states such as dreaming. Recent work argues that Freudian ideas such as the ego, primary, and secondary processing could be usefully recast in terms of hierarchical predictive processing (Carhart-Harris and Friston 2010). Specifically, Carhart-Harris and Friston (2010) argued that the secondary process cognition—which could also be called “ego consciousness”—relates to the functioning of high-level transmodal cortex with the DMN being an exemplary system in this regard. In that work, special emphasis is placed on the DMN constraining activity within limbic and paralimbic systems, a default style of information processing that is likely to underpin the controlled, executive quality of ego-consciousness and the “civilized” behaviors it subserves.

Carl Jung largely accepted the core tenets of psychoanalysis put forward by Freud but began his theoretical divergence by examining and highlighting the importance of the “collective unconscious” (see Jung 1936, 1959). Specifically, whereas constructs such as libido, the “id,” repression, and other defense mechanisms are classically Freudian—being especially pertinent to an individual’s own psychology and experience—Carl Jung argued that much of the unconscious is shared among individuals, which is to say, its character is “collective” or universal in nature. Jung took special inspiration from religious iconography where he highlighted common patterns or themes, which he argued reflect foundational matters pertinent to human evolution and thus, human nature. Jung referred to these common cross-cultural patterns and themes as “archetypes”—basic semantic elements or components from which copies or versions can arise—as in genetic transfer to progeny, where there is mostly conservation (of information encoded within the parents’ genome and “memome” or culture) but also some natural variation.

A brief point of clarification here is how Jung conceptualized his notion of the ‘unconscious’ in contradistinction to Freud. Scholars have begun to question the assumption that Jung developed his notion of the unconscious primarily in opposition to Freud, suggesting that his conception of the unconscious was likely derived from a deeper and common lineage with 19th-century German Romantic Philosophy, especially of the *Naturphisosophen* tradition (Balenci 2022, Clark 2023). Indeed, Jung himself noted affinities between his own conception and that of scholars such as Schelling and Carus (Jung 2014a, p. 3, Jung 2014b, p. 515). In contradistinction to Freud’s more retrospective and reductive notion of the unconscious, these earlier notions conceptualize the unconscious as serving a future-oriented and creative function, which has been described as prospection-oriented and ripe with unactualized potential (Rogers 2020). Indeed, recent treatments of such traditions point to their overlap with Jung’s emphasis on mythological imagery and symbolism (McGrath 2012, Cambray 2017, Barentsen 2020, Clark 2023). Most important for our thesis, this notion of the unconscious confers a more natural overlap with phenomenology of psychedelics, wherein encounters with meaningful and prospective insights are a predominant feature (Gandy et al. 2022, McGovern et al. 2024, Kugel et al. 2025). On this view, the German Romantic lineage offers a more accurate intellectual genealogy of Jung’s thought, and a richer framework for understanding how unconscious content manifests during altered states.

Jung called the archetypes ‘archaic images’ implying that they generally express in the visual or imagistic modality and that they are evolutionarily ancient, being preserved across many generations, effectively canalized into the human psyche (Carhart-Harris et al. 2022); why? Because they are recurrent salient aspects of our character as a species. Another important distinction between Freudian and Jungian psychology is the emphasis Jung placed on spiritual experience and its relationship to the emergence of the unconscious into consciousness. Indeed, Jung believed his psychology better reflected the quality and capacity of the human psyche that transcends the ego, and he argued Freud placed excessive emphasis on sexuality and negative, e.g., aggressive qualities of the psyche.

Thus, in assuming a broader range of themes and motifs to human cognition and behavior, Jung argued beyond the notion of a personal unconscious, for a collective unconscious that was universal and a product of a common, inherited past (Jung 1912, 1919). Prototypical notions of the collective unconscious were initially outlined in Symbols of Transformation (1912). The idea of the term “collective unconscious” was iterated further in Instincts and the Unconscious (1919) and then expanded on in lectures in 1936 (Jung 1936), *Archetypes and the Collective Unconscious* (1959), and in a lecture called “*The Concept of the Collective Unconscious*” (Jung 1936). Jung argues that aside from the individual unconscious, there are inherited patterns of thought common to all humans. He writes:

*“My thesis then, is as follows: in addition to our immediate consciousness, which is of a thoroughly personal nature and which we believe to be the only empirical psyche (even if we tack on the personal unconscious as an appendix), there exists a second psychic system of a collective, universal, and impersonal nature which is identical in all individuals. This collective unconscious does not develop individually but is inherited. It consists of pre-existent forms, the archetypes, which can only become conscious secondarily and which give definite form to certain psychic contents”—*Jung, *Archetypes of the Collective Unconscious,* 1959, p. 43*.*

It should be noted here that there are more and less deflationary interpretations of the collective unconscious. A more deflationary account is that the collective unconscious simply corresponds to the fact that human brains have predispositions about the types of mental models they would form, based on what is common across human populations through evolutionary history (Gooch 1972). More elaborate, metaphysical accounts consider the collective unconscious as being a means of accessing some type of divine aspect of being (see Cook 1987). We work with the more deflationary account that the collective unconscious as simply a propensity to form specific internal psychological patterns and imagery based on commonalities across ancestral populations that share the same (cultural) eco-niche and thereby come to share the same generative models of their lived world.

### The social brain and archetypal stories in the collective unconscious

Extending on Freudian formulations, Jung suggested that human motivation to action was drawn from a broader set than aggression and sex or “thanatos” and “eros” (Freud 2001).

The (collective) unconscious, Jung proposed, consists of symbolism of basic life processes (e.g. ‘Birth, Initiation’), and social roles (e.g. ‘Mother, Father, Child’*).* Jung further proposed that such mental imagery was represented in mythology and folklore, as well as in dreams (e.g. Undirected Thinking—see *Psychological Types*, Jung 1921, pp. 20–50). Jung labelled these mental symbols “archetypes” and proposed that they are universal to all humans.

*“Just as the human body represents a whole museum or organs, each with a long evolutionary history behind it, so we should expect to find that the mind is organised in a similar way. It can no more be a produce without history than is the body in which it exists… I am referring to the biological, prehistoric, and unconscious development of the mind in archaic man, whose psyche was still closer to that of an animal”* (Jung 1959*,* p. 64)*.*

One issue, which requires brief elaboration, is the notion of inheritance. It is sometimes interpreted that inheritance here means a kind of genetic inheritance (see Goodwyn 2020). However, Jung noted in later work that this is not what he meant and instead noted that archetypes would arise *via* complex reciprocal interaction between natural selection, cultural, and learning processes (see also Becker and Neuberg 2019). Moreover, the notion of archetypes is often misinterpreted as being specific to a particular image (e.g. the mother archetype being consciously imagined as representing one’s actual mother). On this, he noted:

*“The term ‘archetype’ is often misunderstood as meaning certain definite mythological images or motifs. But these are nothing more than conscious representations; it would be absurd to think that such variable representations could be inherited. The archetype is the tendency to form such representations of a motif- representation that can vary a great deal in their detail without losing their basic patterns. There are, for instance, many representations of the hostile brethren, but the motif itself remains the same (*  Jung and von Franz 1964*,* p. 58).

Thus, while the specifics of the representation are not inherited, the basic motif (i.e. archetype ‘as such’) is. Recent work has reformulated archetypes in accordance with modern advances in evolutionary psychology, niche construction and cognitive science (Creanza and Feldman 2014, Laland et al. 2016, Heyes 2018, Badcock et al. 2019, Vaughn Becker and Neuberg 2019, Veissiere et al. 2019, Vasil et al. 2020, Krafft et al. 2021).

This work (Vaughn Becker and Neuberg 2019) describes archetypes as such arising from core social goals and motivations that characterized ancestral social environments. According to this view, archetypes can be characterized as “core representations of social instincts: dynamic patterns of perception, memory, and action that develop ontogenetically (they can take culturally and individually variable forms) within phylogenetically sculpted channels that preserve a species typical form” (Vaughn Becker and Neuberg 2019, p. 59). Eager readers will recognize the relevance of “canalization” to this description of “phylogenetically sculpted channels” as the two are synonymous (Carhart-Harris et al. 2022). Whilst archetypal representation likely reflects the social–cognitive challenges faced by ancestral populations (where challenges are evolutionary pressures that drive natural selection, and thus, phenotypic canalization), variance of life experiences necessitates them being crucially dependent on experience (see Cosmides 2000, Becker and Neuberg 2019).

Complementary work has aimed to recast archetypes under the rubric of the FEP. This work suggests archetypes correspond to internal representations or mental simulations of recurrent social patterns and species’ typical motivation across evolution (McGovern et al. 2025). These accounts share the perspectives that archetypal representations may arise as a function of specific survival imperatives (e.g. avoid being killed by hostile beings), resulting in social–motivational systems (e.g. avoiding hostile beings), resulting in a propensity for humans to evolve psychological (and neurological) templates enabling optimal navigation of these threats (e.g. hostile or malevolent archetypes such as the monster or beast, trickster, or witch; Hess et al. 1997  Marsh et al. 2005, Neuberg and Schaller 2015; McGovern et al. 2025). We touch on the mechanisms of how archetypes (i.e. archetypal stories) emerge below.

### Synthesis: the default mode, archetypal transmission, and archetypal story

The claims advanced by recent work on archetypes (Vaughn Becker and Neuberg 2019) are commensurate with the notion that brain activity (as a hierarchical inference) gives rise to the emergence of archetypes. Much research supports the notion that the brain integrates sensory data and creates generative models of the world through recursive and ongoing sensory feedback and initiates predictive simulations based on this feedback (Mesulam 2002, Buckner et al. 2008, Hassabis and Maguire 2009, Yeshurun et al. 2021). This multimodal information descends the subcortical–cortical hierarchy and becomes more concrete (and less abstract) the closer the proximity to sensory regions. *Via* this ongoing sensory feedback, the domain-general cortex is thought to impose high-level (more abstract) priors on sensory input (Parr et al. 2022). If we accept the generative model may be differentially susceptible to updating as a function of developmental stage (e.g. an infant primarily motivated toward attainment of food and safety, differentiating their mother from stranger, or an adolescent prioritizing social acceptance)—and that domain-general regions generate descending predictions for sensory regions—then it is plausible to imagine that archetypal scripts may be encoded *via* reciprocal and recursive exchange with external milieu.

If forced to offer a mechanistic hypothesis, it seems natural to speculate that archetypal ‘images’ and ‘stories’ will be encoded in the brain’s functional architecture, with archetypal ‘stories’ more likely to be encoded in temporally slow trajectories, see Peill et al. 2024, for example. The representation of archetypal ‘images’ may depend on a flip in information flow, away from the ordinarily dominant constraining, entraining, and compressing top–down flow to something more anarchic and free-flowing. Thus, our thesis could be stated as ‘archetypes manifest when the high-level cortex and the lower-level cortex exchange information more freely and with greater parity’. In terms of a functional marker of this effect, an expanded repertoire of brain states including lower as well as higher-frequency spatiotemporal wave patterns (harmonics) traversing the brain’s various hierarchical levels, may be a promising candidate marker or index to empirically explore (Atasoy et al. 2017). Another would be the so-called “collapse” of the brain’s functional gradient (Girn et al. 2022), decreased top–down and increased bottom–up information flow—as seen through altered travelling wave patterns, for example (Alamia et al. 2020), and increased fine-grained (and decreased coarse-grained) spatiotemporal patterns in brain activity (Atasoy et al. 2016, 2017, 2018, Siegel et al. 2024). There is another possibility, however, which is the transient recovery of constraint or organization in brain activity within the psychedelic state, “pushing back” against an otherwise entropic background. This could be construed as unstable attractors, like saddle points, where archetypal images not only form but also readily fragment or change. It is a working hypothesis, motivated by preliminary data pertaining to DMT “entity encounters” that such transient “negentropic” moments underlie the emergence of complex visions such as DMT entity encounters (Timmermann et al. 2019).

It is a central position of this piece that archetypes emerge because of recurrent evolutionary pressures, and associated patterned sensory encounters and behavioral practices, through human phylogeny (see below). In a sense, the general theme of learning and the evolutionary construct of “canalization” bears some relevance in this regard. We also propose that the manifestation of these simulations occur *via* the mechanisms of hierarchical generative models (see Kiefer and Hohwy 2018, Ramstead et al. 2022). These representational systems are experientially dependent and emerge due to a complex interplay between innate (and thus, evolved) cognitive phenotypes and (developmentally contingent) environmental feedback reinforcing when serving beneficial adaptation, in keeping with principles of natural selection.

Or, in terms of variational free energy minimization, Bayesian model selection (Frank 2012, Campbell 2016, Da Costa et al. 2021, Vanchurin et al. 2022, Friston et al. 2023).

We note that the specific content of archetypal representations naturally varies according to cultural context, but the same underlying themes are apparent (see Vaughn Becker and Neuberg 2019), i.e. in their most basic form, archetypes are universal and culturally independent.

### Social–motivational attractors: fundamental motivators as attractor states in development

As alluded to above, archetypes may develop, at least in part, as a function of the motivational imperatives during development (Vaughn Becker and Neuberg 2019). Attention is a crucial modulator of model building (Feldman and Friston 2010), where it modulates the precision weighting or influence of feedforward information, such as increasing the salience of attended-to sensory information. Precise sensory data, when propagated up hierarchical levels, are more likely to influence or update model parameters and representations (Feldman and Friston 2010, Parr and Friston 2018, 2019). This is well reflected in work that finds that more emotionally salient stimuli are more likely to solicit a shared neural response among a crowd (Schmälzle et al. 2015). Even at high levels of the brain’s functional hierarchy, there is evidence that higher attention paid toward different stimuli can solicit a more pronounced synchrony in brain signaling between conspecifics in higher hierarchical levels (Cohen and Parra 2016, Ki et al. 2016).

#### Toward a shared narrative

An important implication of early attentional propensities is that they allow a construction of a shared reality, in which interacting agents communicate and interact from a relatively common thematic encyclopedia or story book (Campbell 2008). From early in development, children seek understanding and to comply with normative behaviors (Haun et al. 2014, Köster et al. 2020), a mechanism through which downward causation is likely to be learned within the brain, mind, and behavior (Freud 1923). As such, the attentional patterns and motivation toward normative or valued behavior are particularly consequential for the types of information that would comprise an adaptive internal model indexing frequent and valued occurrences of the social world.

Consistent with this, recent functional near-infrared spectroscopy work observes coupled responses in the prefrontal cortex between caregivers and infants (Andrews-Hanna et al. 2010, Raichle 2015, Buckner and DiNicola 2019), as well as older children (Miller et al. 2019). In adulthood, the DMN is an important hub for remembering and imagining (motivationally relevant) events, important for planning and enacting adaptive behaviors (Sambuco et al. 2022). The DMN thus seems to support narrative building and social action between conspecifics. In turn, this enables the internal simulations of recurrent social themes and motifs, the kind that constrain generative models (i.e. the set of predictions descending from higher brain regions).

Enactment of these generative models in action selection may result in less surprise (prediction error) and more confidence (precision weighted priors) in social exchange and perhaps help conspecifics reduce each other’s prediction errors, favoring alignment (de Bruin and Michael 2021) or attunement (Stern 1985).

An emergent question here pertains to what types of behaviors or encounters enable organisms to converge on a shared narrative or set of assumptions and representation in social exchange. In recent work, the notion of asymmetric information flow (i.e. when one social agent has more precise priors than another based on experience) is leveraged to explain how expectations attune attentional patterns. This type of information flow is thought to characterize many interactions between (for example) adults and children (see Vasil et al. 2020). Consistent with principles of hierarchical predictive processing, evidence from experimental and computational work suggests that expectations (priors) in older adults are relatively more precise than younger adults and children (Wolpe et al. 2016, Karmali et al. 2018). This suggests that (particularly older) adults have relatively more precise priors (more constraining and “rigid” beliefs) and ascribe relatively less precision to incoming sense data (Moran et al. 2014). In iterant couplings between infants/children and adults, the prior beliefs of more inexperienced conspecifics will dominate and influence those of the less experienced, with a biased directionality toward alignment (Friston and Frith 2015). This coupled and recurrent asymmetric information flow naturally imbues the inexperienced conspecific with the ability to learn the kinds of narrative and information that make up a generalized and generative model (see also Boyd and Richerson 1988). In turn, this allows learning and integration of recurrent social motifs, and behavioral scripts characterizing their eco-niche. One can think of such evolutionary learning as a “propagating facture” (Azarian 2022), iteratively adding depth to canalized phenotypes over generations. Interestingly, recent work characterizes psychedelic experiences as opening a kind of critical period for adults, wherein the dynamic of relaxed beliefs coupled with the more precise (and hopefully adaptive; see McGovern et al. 2024) input of conspecifics, allows model revision (Nardou et al. 2023).

With respect to how this evolutionary learning process influences belief updating in coupled agents—attunement to the (social and environments) niche instantiates a kind of synchronization of prior beliefs (i.e. each other’s mental states)—such that the structure and dynamics of embodied brains recapitulate the structure and dynamics of the social niche in which they find themselves. This is well captured by theoretical work that shows how endowing two coupled hierarchical dynamical systems with an expectation to infer hidden causes that generate other agents’ actions, which enables a mutual informational flow that serves to synchronize statistics of their prior beliefs (Friston and Frith 2015). In the case of two communicating agents, prior beliefs are thus used to recognize canonical behaviors generated by oneself and others (see Friston and Frith 2015, Constant et al. 2018). This is substantiated by a series of hyper scanning (i.e. interacting brains scanned at the same time), which shows the synchronizing effects of cooperative communication between older and younger conspecifics (Stephens et al. 2010, Pérez et al. 2017, Nummenmaa et al. 2018, Liu et al. 2024). As such, environments with more and less experienced social actors allows younger conspecifics the ability to learn the value and of behavioral scripts and action policies that enable convergence to shared narrative and common representations (Ramstead et al. 2016, Renzi et al. 2017, Veissière et al. 2020).

Interestingly, the degree of interbrain synchrony can predict subjective meaningfulness of communication (Stolk et al. 2014, Bilek et al. 2022), recall accuracy of communicative encounters (Zadbood et al. 2017), and emotional resonance with political speech (Hasson et al. 2012, Hasson and Frith 2016, Schoot et al. 2016, Stolk et al. 2016). This suggests that synchronous brain dynamics map to attuning attentional policies—enabling increased parsimony and overlap in the kinds of generative models held by communicative social agents. More generally, this process facilitates initially chaotic social dynamics to converge on a set of attractor representations, allowing for a generally shared narrative. The psychological construct of “communitas” is relevant in this regard (Kettner et al. 2021).

Individual agents can thus instantiate their models *via* a brain architecture that is sensitive to information learned over different temporal scales, enabling emergence of time-invariant (i.e. how to forage for food) and time-variant (i.e. how one greets others according to sociocultural niche) social motifs and action policies. As such, the archetypal stories and narratives may arise partially due to this iterative communicative process, coupled with the hierarchically instantiated architecture of the brain.

### Synthesis: development and convergence to a collective minima

In this section, we have outlined evidence that infants have attentional regimes that preference the type of information important to ancestral populations. This work suggests that the morphology and core motivational needs of human, throughout our extended ontogeny (Sterelny 2012), preference social information. In turn, this allows for the learning and integration of socially adaptive information. This is reflected in the brains of infants in both perceptual tasks and in coupled dynamics with other embodied brains. This learning and integration process depends on a hierarchically structured neural architecture. Through systems such as the DMN simulating recurrent social patterns (Mars et al. 2012, Xie et al. 2016), the brain can learn to integrate and represent useful and adaptive information. The process of multiple embodied brains learning information in the service of evolutionary imbued social goals (e.g. ‘seeking a caregiver, avoiding predators’) allows the generation of shared narratives and representations. Archetypes can therefore be seen as canalized motifs—that have been canalized through recurrent social encounters that carry salience for the given species. For example, human infants, with their extended ontogeny and exceptional vulnerability and dependence on others, must be able to model the notion of a caregiver to discern who serves their survival and well-being ‘versus’ who does not or who may intend harm toward them.

## Discussion

**Figure 1:**
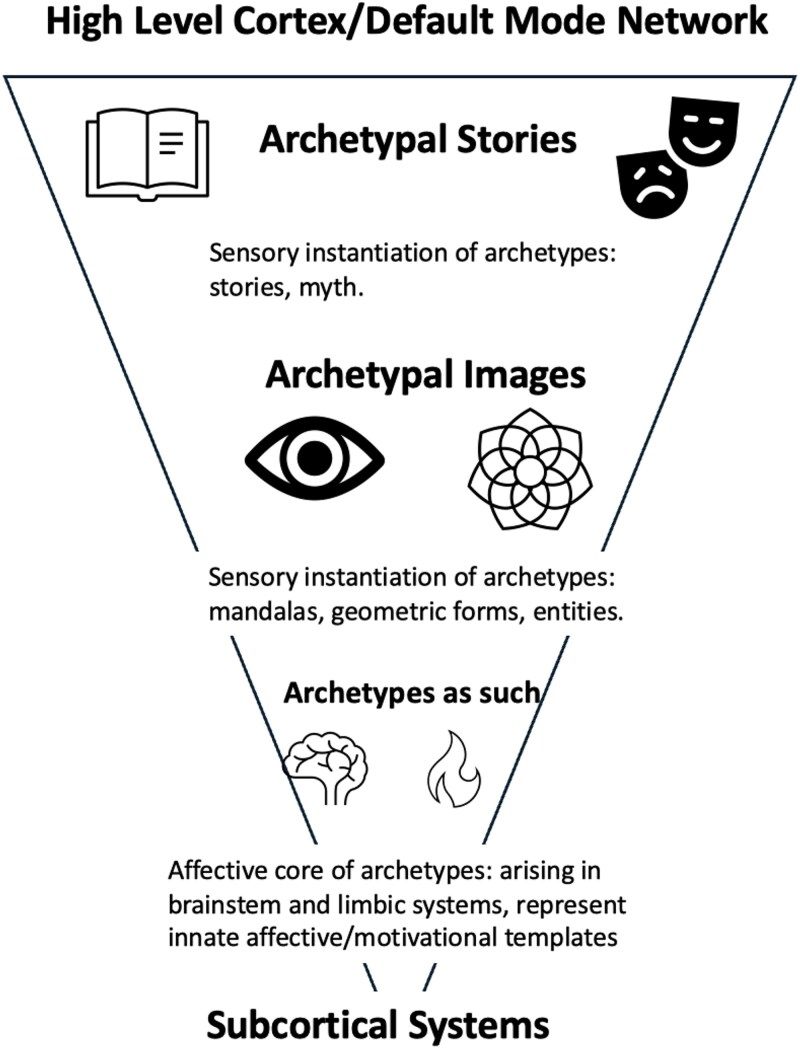
Depiction of our trilogical interplay model, depicting archetypes as such, archetypal images, and archetypal stories within the context of the hierarchically instantiated systems in the brain, ranging from high-level networks to subcortical systems.

We have endeavored to explain how Jungian ideas, particularly of the collective unconscious and archetypes, can be reconciled with the latest neuroscientific accounts of brain function. Whilst this is an ambitious synthesis, we have suggested that there is growing evidence that enables us to begin such a project and point toward empirical investigations for deepening our understanding. Our synthesis, we suggest, carries two primary implications. First, archetypal representations are likely a reflection of social imperatives and motivations enabling resolution of recurrent adaptive challenges (e.g. an infant being able to distinguish caregiver from non-caregiver) and behavioral scripts facilitating their resolution (e.g. a hero’s journey or coming of age process enabling acceptance in the local socio-cultural niche). Second, these representations naturally emerge from the notion of the brain as an inference engine, with cascading predictions facilitating the emergence of archetypes as such, archetypical images, and archetypal stories or narratives (see Fig. 1 for overview). We mean “hierarchical” in the sense that hierarchically higher regions (a.k.a., domain general regions such as the high-level transmodal association cortex) coarse grain (*via* downward causation) information processed by domain-specific regions (e.g. sensory cortex)—enabling an effective simulation of the world or “controlled hallucination” (Seth 2021)—as sharp in fidelity as it may seem.

We propose that during archetypal visions such as those occurring under psychedelic drugs, this hierarchical divergence in information passing and processing collapses such that high- and low-level cortices can communicate more freely. We also speculate that this communication expresses as an extended repertoire of brain states including increased activation of higher frequency spatiotemporal patterns or harmonics (Atasoy et al. 2017) that more easily traverse the (distributed) functional hierarchy. We also hypothesize that this anarchic quality to brain function is indexed by greater bottom–up and less top–down information flow (Alamia et al. 2020), and finally, we encourage an examination of high frequency harmonics within the ventral visual stream, with more information flow across hierarchical levels therein (e.g. between medial temporal structures and V1), as another promising candidate marker of archetypal visions under psychedelics, or other inducers for that matter.

Returning to the psychology, construction of archetypal motifs allows agents to construct shared narrative of increasing abstraction and temporal depth (e.g. one’s initial representation of mother is your biological mother or primary caregiver but then becomes a more abstract and temporally invariant as one samples more instances of mother: resulting in an average based on all encounters with mother, and an understanding of how that abstraction differs from non-mothers or fathers). We suggest that (i) the emergence of these archetypal patterns reflects social imperatives/instincts and the types of behaviors that enable or disable their attainment, and (ii) these internal representations arise through two types of reciprocal exchange: one in the cortex between hierarchically higher and lower brain regions, and that between the agent and other social agents. To close, we briefly outline theoretical and clinical implications of our proposal.

This synthesis is multi-faceted, covering depth psychology and human neuroscience, but there are tangible potential applications of it to discrete fields depending upon the emphasis one wishes to draw. For example, a clinician may adopt the view that archetypes correspond to basic narratives on which one rests one’s view of themselves and the world. Working with this assumption in psychoanalysis could bear fruit, as is already done in Jungian analysis. However, this approach could also be incorporated into psychological integration work done after a psychedelic experience, e.g. most obviously as psychedelic therapy (Carhart-Harris et al. 2016). Additionally, a social neuroscientist may wish to understand the neural events (within and between individuals) that signal the establishment and maintenance of shared narratives. In a general sense, then, viewing archetypes through the lens of the hierarchical Bayesian brain representing recurrent social motivation, challenges, and solutions offers the chance for theoretical pollination between discrete fields, as well as a deepening and validation of Jungian theory. Moreover, this proposal offers tangible and testable hypotheses of how archetypal representations can emerge into conscious experience. Further development of these contact points in discrete fields could enable further refinement to core Jungian ideas, primarily to establish construct validity of archetypes, as well as other Jungian ideas.

### Limitations and future directions

It is important to note that we do not argue that all Jungian constructs fare well in light of modern neuroscience. For example, his views on psychedelic use were at times prejudiced and ethnocentric, and his views on schizophrenia do not fare well with modern evidence. Furthermore, there are other concepts of Jungian thought (which are not covered here) which may not stand up to scrutiny. For example, we have not discussed Jungian complexes, thought to originate in the collective unconscious. There is potential for further exploration into complexes. For example, they could be interpreted as erroneous but highly precise predictions that hinder the process of model optimization (alternatively described as canalized pathological beliefs serving as a psychological defense; Carhart-Harris et al. 2022). Alternatively, complexes may be considered as psychological adaptations designed to resolve competing imperatives—such as the need for attachment and authenticity (e.g. due to an attachment figure who can be threatening), resulting in a “negative mother complex”. In this latter case, the issue is less to do with the erroneous prediction and more to do with the fact that there is no clear way to meet or resolve the dispute between these competing imperatives.

This line of research could provide novel insights and have clinical applications. Our analysis also does not extend to dream states, which form a crucial part of psychology and could reasonably profit from integration between Jungian psychology and modern neuroscientific work. Another limitation of the account forwarded here is that aspects of account may be speculative in places. For example, one aspect of our account suggests that subcortical structures may encode “archetypes as such.” While extant work suggests increase in bottom–up signaling, as well as emergence of archetypal imagery under altered states (Carhart-Harris et al. 2014), the notion that archetypes as such are encoded in subcortical structures would require further empirical verification. This could be addressed in future work through different methodological or analytical means. For example, future research could leverage multi-modal imaging to test if recurrent archetypal imagery directly corresponds with activity in midline and limbic structures, including the hippocampus, periaqueductal gray, or basal forebrain. More indirectly, future work could additionally implement techniques such as natural language processes (e.g. semantic embedding or clustering algorithms) to dream reports or reports of psychedelic phenomenology. These reports could then be used to ask if particular archetypes relate to activity in evolutionarily older subcortical regions (e.g. hippocampus, periaqueductal gray, hypothalamus) using techniques such as representational similarity analysis (RSA) or model-based fMRI.

Another prediction our trilogical interplay also gives rise to is the relationship between inhibition of cortical inhibitory networks, brain entropy, and propensity to experience archetypal imagery. We predict that archetypal imagery should principally arise when higher cortical networks are inhibited, reducing their inhibitory function. Likewise, we would also expect that increased entropy in brain dynamics could be an important prerequisite for encounters with archetypal imagery. This could be tested by seeing whether increased frequency and depth of archetypal experiences correspond with both an inhibition of large-scale networks and increased signal complexity (measured with metrics such as the Lempev–Ziv complexity metric).

A more conceptual limitation concerns the fact that Jungian constructs emphasize symbolism rather than attempting to describe mechanistic processes. Given the nature of our synthesis, we must note the inherent risk of conflating metaphor present in Jung’s work with literal operationalization. While we note parallels between neural behavior and archetypes, we note that these should be interpreted primarily in a heuristic sense, rather than as a one-to-one mapping of phenomenology onto brain regions. To avoid such errors, including the mereological fallacy (i.e. attributing psychological properties to neural substrates; Bennett and Hacker 2003), we note that archetypes should principally be understood in terms of emergent and symbolic phenomena, whose appearance in brain activity more likely reflects structural predispositions rather than bespoke encoding. While phenomenology (including archetypal imagery) no doubt corresponds with underlying neural architectures, this should not be read as a mutually causal relationship or as having a direct correspondence. As such, our use of terms such as shared minima or interbrain synchrony is intended to provide a kind of neurophenomenological scaffold for archetypal imagery or experience, rather than providing a purely mechanistic account. This is important, as we aim to preserve the teleological, symbolic, and affective richness of Jung’s accounts of archetypes, whilst also providing meaningful engagement with extant neuroscientific literature. Despite limitations, and appropriate precautions, we propose that our work offers a way in which Jungian archetypes can be more seriously considered and adopted for the purposes of understanding the brain and (particularly its social) function.

## Conclusion

Our work provides a novel interpretation of Jungian archetypes as installed in generative models and are allowed “off the leash” as unstable attractors, e.g. their manifestation can perhaps most clearly be seen under the effects of psychedelic drugs, due to their specific action on the mechanisms of hierarchical predictive processing in the brain. We have also argued that extant evidence from social, theoretical, and developmental neuroscience lends support to the Jungian constructs of the collective unconscious and archetypes. We leveraged evidence from developmental, social, and clinical neuroscience to argue for the validity of these concepts and point the reader toward ways to test specific hypotheses about how archetypes are encoded in brain activity and may be decoded from this activity. In doing so, we hope this synthesis can foster a dialogue between modern neuroscientific and psychoanalytic communities and lend a warranted validation to some important constructs embraced by the latter and traditionally neglected by the former.
